# Rabbit VX2 lung tumor models can form early nodal metastases

**DOI:** 10.1186/s12957-019-1774-6

**Published:** 2019-12-29

**Authors:** Alexander Gregor, Kosuke Fujino, Nicholas Bernards, Tomonari Kinoshita, Yamato Motooka, Terunaga Inage, Tsukasa Ishiwata, Zhenchian Chen, Hideki Ujiie, Chang Young Lee, Kazuhiro Yasufuku

**Affiliations:** Division of Thoracic Surgery, Toronto General Hospital, University Health Network, 200 Elizabeth St, 9N-957, Toronto, ON M5G2C4 Canada

**Keywords:** Disease models, Animal, Rabbits, Nodal metastasis, Lung cancer

## Abstract

**Background:**

The rabbit squamous cell cancer line, VX2, has been used to generate various tumor models in rabbits. It is notable for its ability to generate nodal metastases. However, the timing and extent of nodal metastases vary by primary inoculation site and methodology. The development of metastases specifically in lung cancer models has not been well-described. We sought to characterize the generation of nodal metastases in rabbit transbronchial VX2 lung tumor models.

**Methods:**

Rabbit VX2 lung tumor models were created in the right lung via transbronchial injection and serially imaged by computed tomography. Rabbits (*n* = 15) were sacrificed from between 5 and 24 days post-inoculation for collection of the ipsilateral and contralateral paratracheal lymph nodes. These underwent histopathological evaluation for metastases using hematoxylin and eosin as well as cytokeratin AE1/AE3 immunohistochemical staining.

**Results:**

Nodal metastases were detectable as early as 1 week after inoculation but were more prevalent with longer inoculation; all rabbits at > 2 weeks post-inoculation had nodal metastases. Contralateral metastases were in general seen later than ipsilateral metastases. Lymph node volume did not predict the likelihood of nodal metastases (*p* = 0.4 and *p* = 0.07 for ipsilateral and contralateral nodal metastases, respectively), but primary tumor volume was significantly associated with the likelihood of nodal metastases (*p* = 0.001 and *p* = 0.005 for ipsilateral and contralateral nodal metastases, respectively). Ipsilateral metastases were detectable at a tumor diameter of 1 cm; contralateral metastases were more variable but in general required a tumor diameter of 2 cm.

**Conclusions:**

Rabbit transbronchial VX2 lung tumor models generate nodal metastases relatively early after inoculation. These results suggest such models may be valuable tools in the investigation of novel therapeutic modalities relevant for the treatment of both early-stage and locally advanced lung cancer.

## Background

Lung cancer is the leading cause of cancer death globally [1]. Effective animal models play a key part in developing and evaluating new therapeutic approaches. Mouse lung cancer models are reasonable options for evaluating systemic therapy but have limitations for evaluating other modalities, such as novel surgical and endoscopic technologies. Rabbits, by comparison, are better sized for such research. VX2 is a rabbit squamous cell cancer line that has been used to generate rabbit tumor models at a variety of sites, including uterus, tongue, stomach, hypopharynx, breast, rectum, muscle, cheek, liver, and lung; this cell line is well-documented in its ability to generate nodal metastases [2–11]. However, there is significant variation in the timing of nodal metastases based on VX2 inoculation protocol (e.g., primary inoculation site, cell count of inoculum, media). This is further complicated by heterogeneity in the time from inoculation to sacrifice (ranging from days to months). It is known that VX2 lung tumor models can generate nodal metastases, but this has been largely demonstrated on autopsy after animals died from uncontrolled disease at 26 to around 40 days [12, 13]. Those studies that stratified rabbits by time from inoculation did so for assessing changes on imaging or responses to radiofrequency ablation; the timing of nodal metastasis development was therefore unclear [12, 14]. Considered together, VX2 nodal metastases have not been well-characterized for rabbit lung tumor models. Understanding when VX2 lung tumor models develop metastases and the pattern of spread is critical in informing the use of these models as surrogates for both early-stage and advanced lung cancer patients. Lymph node metastasis is significantly associated with lung cancer prognosis and is a key factor in treatment decision-making [15, 16].

Our group has previously described a peripheral VX2 lung tumor model using bronchoscopic injection [11]. An extracellular matrix (ECM) protein suspension is vital to prevent post-injection leakage, which could otherwise result in unintentionally diffuse disease or expectoration of the inoculum [17]. Compared to previous work characterizing VX2 nodal metastases, the combination of a different inoculation site (lung) and media (ECM suspension), among other differences, may significantly alter the generation of spontaneous VX2 nodal metastases. We sought to determine whether transbronchial VX2 inoculation can generate early nodal metastases (i.e., prior to significant progression of disease) and if so, to characterize the timing and nature of those metastases. We hypothesized that we could identify a time point where the models would have both primary tumors and nodal metastases without development of widely disseminated disease, making them viable models for future study.

## Methods

Animal use and care was previously approved by the University Health Network Animal Care Committee (Animal Use Protocol 4152), in accordance with relevant provincial and federal statutes. Animals were housed in a dedicated facility and cared for by dedicated veterinary staff.

### VX2 tumor inoculation

A stock of VX2 tumor cells was propagated in rabbit hindlimb muscle, as previously described [11]. A total of 16 female New Zealand White (NZW) rabbits (Charles River, St. Constant, QC) weighing 3–3.5 kg were inoculated via bronchoscope, as we have previously published [11]. In brief, following induction of general anesthesia via isoflurane, a size 1 pediatric laryngeal mask (LMA Unique, Teleflex LMA Company, Singapore) was placed. An ultrathin bronchoscope (XP-160F, Olympus, Tokyo, Japan) was navigated to the peripheral right lower lung and 500 μL cell mixture (50% cell suspension in Hank’s Balanced Salt Solution, 50% ECM [Matrigel, Corning, NY]) was injected using a 21-gauge transbronchial needle (NA-2C-1, Olympus, Tokyo, Japan). Eleven rabbits were inoculated with approximately 5 × 10^6^ cells; four rabbits were recruited from a separate study that had been inoculated with 1 × 10^6^ cells. This reduced cell count was due to lower than expected recovery of viable cells from the propagating muscle tumor. One rabbit died in the immediate post-inoculation period prior to anesthetic recovery from a pneumothorax. No imaging or tissue was collected from this rabbit, and thus 15 rabbits total were included in the study.

### Imaging and tissue collection

Starting post-inoculation day 5, serial axial computed tomography (CT) was performed (GE Locus, 80 kVp, 50 mA, 0.15 mm slice thickness, 102 mm field of view) up to 21 days post-inoculation. Rabbits were intermittently removed from the imaging cohort for euthanasia from 5 to 24 days post-inoculation. Sacrifice was performed with induction of anesthesia by isoflurane followed by potassium chloride infusion through the marginal ear vein. Mediastinal lymph node dissection was performed to collect the right and left paratracheal nodes. These nodes are the primary sites of lymphatic drainage for the right lower lobe [18]. The length, width, and height of each collected node and primary tumor were retrospectively measured using the most recent CT data from just prior to sacrifice. The formula for calculating the volume of an ellipsoid is $V=\frac{4}{3}\pi \times a\times b\times c$, representing the radius of the height, length, and width, respectively, as measured based on the axial and coronal slices of maximal diameter. Each rabbit therefore had 3 regions of interest: the primary tumor, the right (ipsilateral) paratracheal node, and the left (contralateral) paratracheal node.

### Histology

Lymph nodes were placed in 10% formalin for approximately 72 h before paraffin embedding and sectioning along the longest axis of the node. Serial sections from the region of maximum nodal diameter were stained with hematoxylin and eosin (H&E) for initial evaluation, as well as cytokeratin AE1/AE3 immunohistochemistry (IHC) for more sensitive metastasis detection. VX2 tumor cells are known to be strongly positive for this marker, while normal nodal tissue is negative [19].

For immunohistochemical staining, slides were deparaffinized with xylene, washed with ethanol, rehydrated in water, and then washed with Tris-buffered saline (TBS). Antigen retrieval was performed by microwaving the slides in 10 mM citrate buffer (00-4955, Invitrogen, Carlsbad, USA) for 5 min. Endogenous peroxidase was blocked using 0.3% H_2_O_2_ in methanol for 30 min. Slides were then washed in TBS with Tween (TBST) and TBS before performing protein blocking using 5% skim milk dissolved in TBST for 45 min at room temperature. Pre-diluted anti-cytokeratin AE1/AE3 (ab961, Abcam, Cambridge, USA) was incubated on the slides overnight at 4 °C. Note that ab961 is purchased pre-diluted and was used at its stock concentration. The following morning, slides were again washed in TBST/TBS and incubated with peroxide-conjugated goat anti-mouse antibody (K4001, Agilent Dako, Carpinteria, USA) at room temperature for 60 min. The slides were washed in TBST/TBS and then exposed to 3,3′-diaminobenzidine-based peroxide reagent (K3468, Agilent Dako, Carpinteria, USA). The color reaction was stopped in water before counterstaining the slides with hematoxylin.

### Statistical analysis

Volumes of the paratracheal nodes and primary tumors were evaluated for their association with the detection of VX2 metastases using the Wilcoxon rank-sum test, with *p* < 0.05 being considered significant. Statistical analysis was performed using *R* (R Foundation for Statistical Computing, Vienna, Austria)*.* Boxplots were created using the package ggplot2 (Springer-Verlag New York, NY, USA).

## Results

**Fig. 1:**
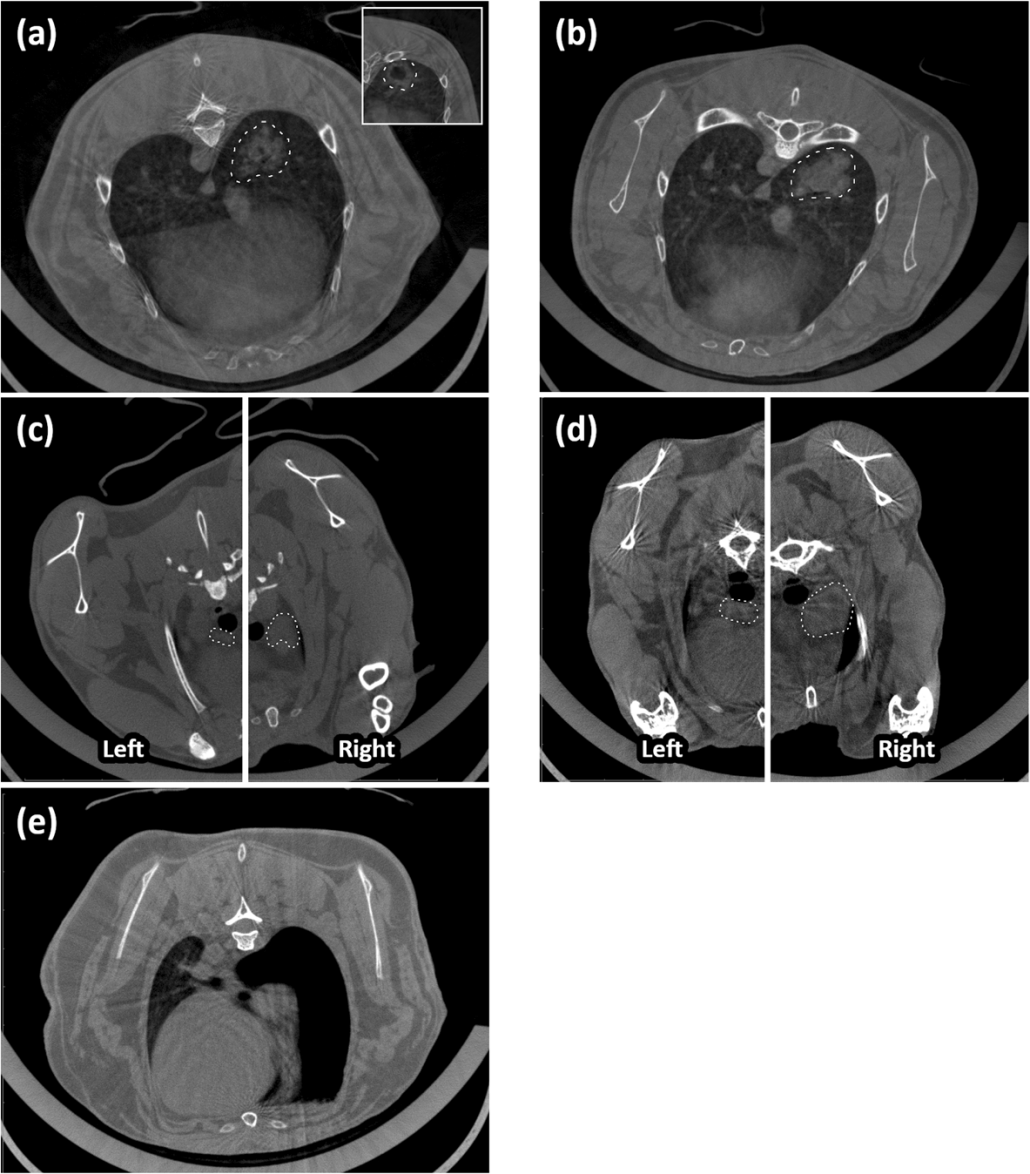
Representative computed tomography images of rabbit VX2 nodal metastasis models. A primary tumor can be seen outlined at 5 days post-inoculation in the right lower lobe (**a**) (although solid in this rabbit, some others had cavitation [inset]). This rabbit’s tumor demonstrated interval growth when re-imaged on day 9 (**b**). Scans from another rabbit at day 5 with negative nodal pathology reveal the left and right paratracheal nodes, which have been respectively outlined (**c**). The left paratracheal node is caudal to the right paratracheal node, requiring two different axial slices to visualize their maximal diameter. Although lymph node size remained relatively stable for the first 2 weeks, progressive enlargement could be more reliably appreciated thereafter. This can be seen in a rabbit imaged at day 21 (**d**). One rabbit developed a bronchopleural fistula with resulting right pneumothorax; the collapsed right lung can be seen lying against the heart (**e**)

**Fig. 2:**
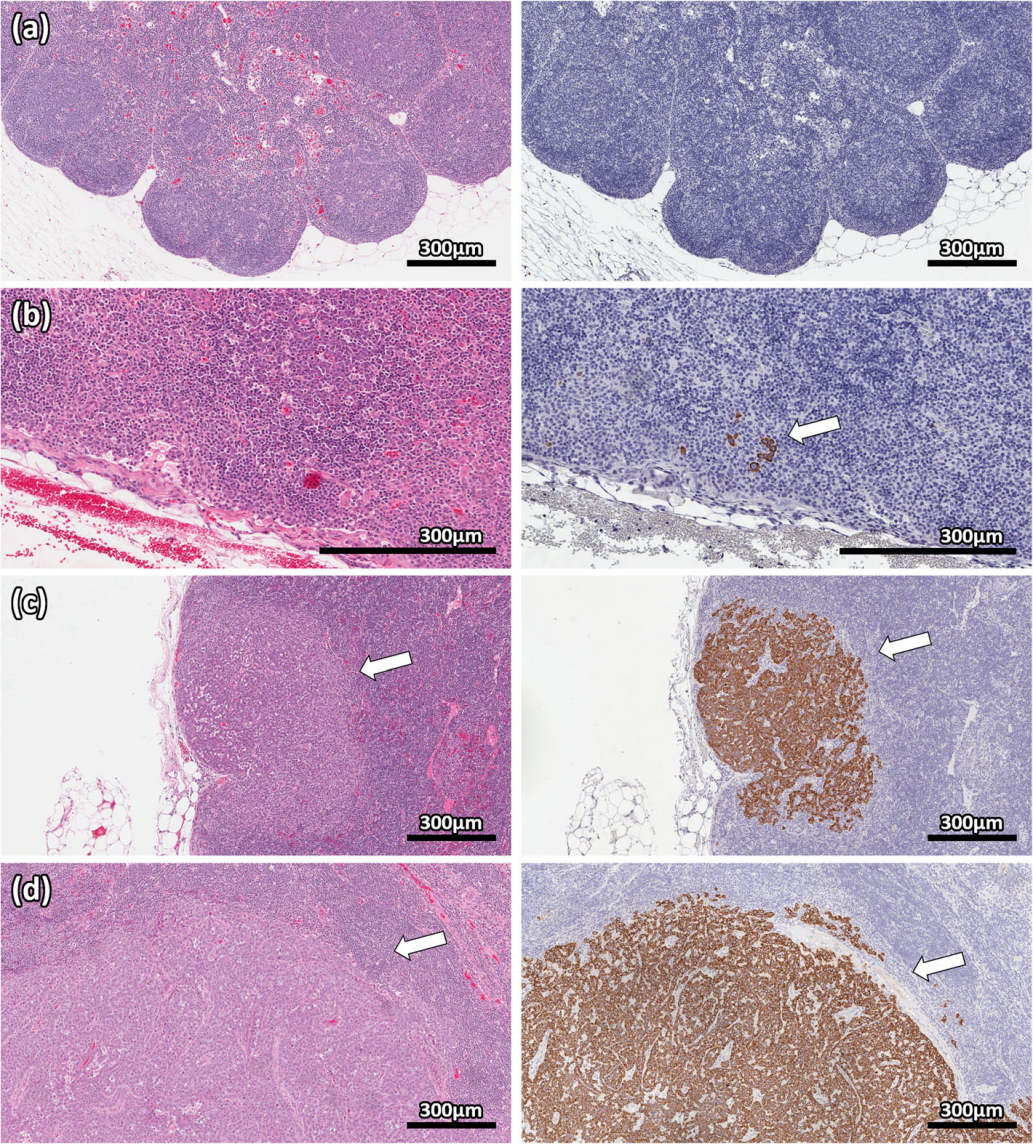
Representative pathology images of VX2 nodal metastases. Hematoxylin and eosin (left), and cytokeratin AE1/AE3 immunohistochemistry (right) stains demonstrate negative nodal tissue (**a**), isolated tumor cells (**b**), micrometastases (**c**), and macrometastases (**d**). The isolated tumor cells were not easily visualized by standard hematoxylin and eosin staining but were clearly seen on immunohistochemical staining. VX2 cells have strongly positive cytoplasm on immunohistochemistry. Scale bar represents 300 μm

VX2 tumor inoculation was successful in all 15 rabbits. Review of CT images consistently demonstrated a solitary mass in the right lower lobe (Fig. 1a) that progressively increased in size over time (Fig. 1b). There were no obvious changes in lymph node appearance in the initial 2 week period after inoculation (Fig. 1c); however, increasing size became apparent at later time points (Fig. 1d). Longer-term inoculation increased the likelihood of detecting nodal metastases using IHC (Table 1). All metastases detected at ≤ 7 days inoculation were classified as isolated tumor cells (ITCs; i.e., AE1/AE3^+^ deposits < 0.2 mm in size); the one rabbit with ITCs in the left (contralateral) paratracheal node also had ITCs in the right (ipsilateral) paratracheal node. From 8 days onward, all metastatic deposits were classified pathologically as micrometastases (0.2–2 mm) or macrometastases (> 2 mm). Representative images demonstrating negative lymph nodes, ITCs, micrometastases, and macrometastases are shown in Fig. 2.

**Table 1:** Nodal metastases in the right and left paratracheal nodes, by duration of inoculation

	Days from VX2 tumor inoculation to sacrifice			
	≤ 7 days	8 to 14 days	15 to 21 days	> 21 days
Right paratracheal (ipsilateral)	2/4	4/6	4/4	1/1
Left paratracheal (contralateral)	1/4	0/4*	4/4	1/1

*Left paratracheal nodes were not clearly identified on autopsy in 2 rabbits

**Fig. 3:**
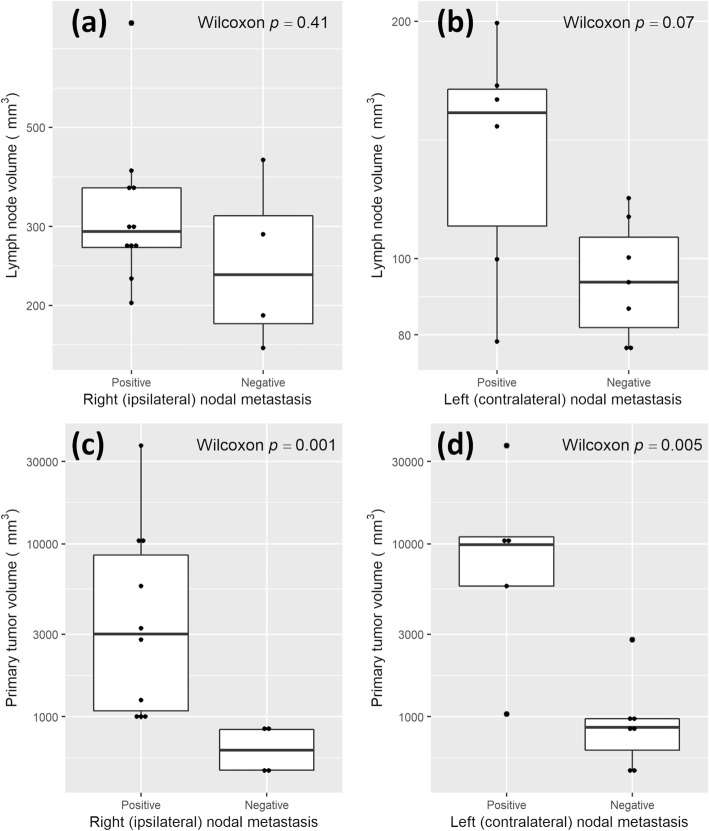
Relation between nodal metastasis status, lymph node volume, and primary tumor volume. There was no statistically significant difference between nodal metastasis status and lymph node volume for the right (**a**) and left (**b**) paratracheal nodes. There was, however, a statistically significant difference in nodal metastasis status and primary tumor volume for the right (**c**) and left (**d**) paratracheal nodes. Note that the *y* axis is displayed in a logarithmic scale to minimize distortion of data by outliers

There was no statistically significant difference in lymph node volume and the presence of VX2 metastases for both the right (*p* = 0.41) and left (*p* = 0.07) paratracheal nodes, although positive nodes tended to be larger (Fig. 3a, b). Note that the left paratracheal node was not successfully identified in two rabbits from the 8–14-day cohort and therefore these rabbits were excluded from analysis of left (contralateral) metastases. By comparison, the volume of the primary tumor was associated with detection of VX2 metastases for both the right (*p* = 0.001) and left (*p* = 0.005) paratracheal nodes (Fig. 3c, d). Accurate primary tumor volume was not available for 1 rabbit due to development of a bronchopleural fistula from the tumor with associated pneumothorax, requiring sacrifice as a humane endpoint (Fig. 1e). This rabbit’s primary tumor data was therefore excluded from primary tumor volume analysis. However, lymph node measurements and tissue from both paratracheal nodes were still able to be obtained and included in lymph node volume analysis. For rabbits with right (ipsilateral) paratracheal nodal metastases, primary tumors were ≥ 960 mm^3^ (equivalent to a 12 mm diameter sphere). For left (contralateral) paratracheal nodal metastases, the primary tumor volume cut-off was less clear given outliers in both the node-positive and node-negative groups. Excluding these outliers, contralateral metastases were detected starting at approximately 5700 mm^3^ (equivalent to a 22 mm diameter sphere). This is a conservative estimate, however, and the outliers would support that contralateral metastases develop with smaller primary tumors.

## Discussion

Despite the differences unique to transbronchial rabbit VX2 lung tumor models (i.e., lung injection, use of ECM media), it appears these models can generate nodal metastases relatively early after inoculation. We found that primary tumor size predicted the likelihood of nodal metastases; however, we recognize this may be a confounder for time from inoculation. Further investigation on whether tumor size has an independent effect on nodal metastasis is needed. By comparison, we were somewhat surprised that lymph node volume was not associated with the likelihood of metastases. This may be related to initial reactive lymphadenopathy from the bronchoscopic procedure, which enlarged early “negative” nodes and thus reduced our ability to detect statistically significant differences. Although metastases at later time points demonstrated large macrometastases, this subgroup was too small to generate significant results. An argument could be made to extend observation beyond 21 days to increase the number of animals with large nodal metastases, but in practice this would prove difficult. The longest surviving rabbit, at 24 days post-inoculation, demonstrated increasingly rapid interval tumor growth between scans and already had complete replacement of the right lower lobe with tumor by the time of autopsy. Such animals are under significant physiological stress that makes them poor models for investigating new technologies and techniques, given their questionable ability to tolerate additional procedures. Nonetheless, we expect that had we access to a larger cohort of long-inoculated rabbits, a clearer correlation between lymph node size and likelihood of nodal metastasis would have been detected. On the opposite extreme, we have not definitively identified the earliest time point at which nodal metastases develop, as even in the earliest cohort (≤ 7 days) we could detect lymph node metastases. Nonetheless, the reduced yield of nodal metastases in this early cohort would suggest that lymph node metastases likely first begin appearing around 5–7 days post-inoculation.

Relating these results to previous reports on VX2 nodal metastases is difficult given the heterogeneity of prior work. The timing of nodal metastases can vary by primary inoculation site. Xu et al. reported that VX2 endometrial cancer models only developed histologically-proven metastases at 21 days [2]. By comparison, there are reports of auricular cancer models developing nodal metastases within 7 days [20]. Local tissue environments, including oxygen tension, vascularity, and tissue compliance, may contribute to these differences. Focusing on only lung VX2 models highlights further differences. In three studies that employed VX2 lung tumor models and reported on nodal metastases, one study injected a cell suspension without ECM under CT guidance, and one performed transbronchial injection of a cell suspension with ECM under X-ray guidance but with a much larger inoculum than used in this study, and one implanted a 1-mm^3^ piece of minced VX2 tumor directly into the lung under CT guidance with no reported cell count [12–14]. Those studies that most clearly identified the time of nodal metastasis relied on autopsy after rabbits expired from disseminated disease without intervention, ranging from 26 to 40 days post-inoculation [12, 13]. The timing of nodal metastasis detection in imaged and/or treated rabbits was less clear, but in general seem to have been detected at 4 weeks or more after inoculation [12, 14]. We have shown here that rabbits develop nodal metastases well prior to these time points. Our data suggests the use of ECM did not impair the ability to form early nodal metastases, even before the 2-week period in which ECM is usually resorbed [13]. This may be related to our use of ECM suspension during intramuscular propagation, which could select out cell populations that more easily breakdown ECM. That slight modifications to VX2 propagation may alter nodal metastasis patterns has been previously shown by Kim et al., who found that altering their VX2 intramuscular passaging method (by processing the lymph node rather than the primary tumor) improved their nodal metastasis rate over time [19]. A final key differentiating factor of our study was the routine use of IHC for all lymph nodes to evaluate for metastases, which improved detection of small tumor deposits that have a higher likelihood to be otherwise missed on routine H&E alone (particularly ITCs).

There are some caveats to this study. The timing of the CT scans was not consistent for all rabbits, resulting in insufficient longitudinal data to generate accurate growth curves. These growth curves may have been an additional predictor of nodal metastases, including the ability to account for any baseline variability in nodal size. Contralateral paratracheal nodal metastases must be understood in the context of rabbit mediastinal lymphatic anatomy, which differs from humans. Subcarinal lymph nodes are inconsistently present in NZW rabbits, which presumably contribute to a propensity for bilateral mediastinal involvement [18]. The bilateral ITCs seen in one rabbit at ≤ 7 days inoculation may be a reflection of this phenomenon. We also recognize that our overall sample size is small; we have attempted to compensate for this through use of non-parametric statistical tests. Finally, our pathological evaluation used slides from the region of maximal diameter, rather than from the entirety of the lymph node. It is therefore possible our results underestimate the timing of nodal metastasis development.

## Conclusion

We have demonstrated that rabbit VX2 lung tumor models can reliably generate nodal metastases. Importantly, this occurs relatively early after inoculation compared to previous experience with VX2 tumor models. Transbronchial injection of VX2 tumor cells in rabbits may represent a valuable model for the investigation of novel therapeutic strategies intended for both early-stage and locally advanced lung cancer, depending on the timing after inoculation.

## Data Availability

The datasets used and/or analyzed during the current study are available from the corresponding author on reasonable request.

## Abbreviations

CT: Computed tomography
ECM: Extracellular matrix
H&E: Hematoxylin and eosin
IHC: Immunohistochemistry
ITC: Isolated tumor cells
NZW: New Zealand White
TBS: Tris-buffered saline
TBST: Tris-buffered saline with Tween
